# *In vivo*-like Organotypic Murine Retinal Wholemount Culture

**DOI:** 10.3791/1634

**Published:** 2010-01-11

**Authors:** Sebastian Gustmann, Nicole Dünker

**Affiliations:** Institute for Anatomy, Department of Neuroanatomy, University of Duisburg-Essen

## Abstract

Targeted ablations of genes and analysis of animal models is the classical strategy for enrolling specific retinal gene function. However, transgenic, retina-specific or conditional knockout mouse models often display early lethality or suffer from severe malformations, preventing an analysis beyond embryonic or early postnatal stages.

Primary cell culture is an alternative to investigate the effects of exogenously applied recombinant factors, overexpression of genes or siRNA-mediated gene knockdown in a controlled environment. Dissociated cell culture has the advantage that the endogenous signals reaching the target cells are reduced, thereby facilitating the identification of exogenously triggered effects after pharmacological manipulation. However, important cell-cell interactions are initially destroyed by enzymatic digestion or mechanical dissociation, even if re-aggregated retinospheroid cultures^1^ are used.

By contrast, organotypic retinal wholemount cultures provide a system close to the physiological *in vivo* situation with neuronal interactions and connections still preserved^2-5^.

In this video article we provide a step by step demonstration of (1) the establishment of *in vivo*-like organotypic retinal wholemount cultures including dissection peculiarities of embryonic, postnatal and adult murine eyes and (2) a dissociation and cytospin procedure for analysis of neuronal apoptosis and retinal cell proliferation in organotypic wholemounts, e.g. after culture in the presence of exogenously applied recombinant factors.

**Figure:**
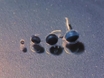


## Protocol

All equipment and reagents have to be purchased sterile or needs to be heat or steam sterilized or sterilized with 70% ETOH.

The authors state that experiments on animals were performed in accordance with the European Communities Council Directive (86/609/EEC), following the Guidelines of the NIH regarding the care and use of animals for experimental procedures and the regulations set forth by the Institutional Animal Care and Use Committee (IACUC) at the University of Duisburg-Essen (Germany).

### Part 1: Enucleation of murine eyes of different developmental stages

#### Enucleation of embryonic eyes

1. Time pregnant matings are set up and the morning of the day on which a vaginal plug is detected in females mating is designated gestation day 0.
2. The pregnant female is sacrificed by cervical dislocation when the development of the embryos has reached the desired stage (here: embryonic day (E) 15) and fixed on a wax board^6^.
3. The abdominal wall is moistened with 70% ETOH, cut along the midline and the skin flaps are fixed laterally by pins^6^.
4. The uterusses are removed from the abdomen, detached and transferred to a beaker with cold PBS^6^.
5. The embryos are separated, transferred to a Petri dish and uterus wall and embryonic membranes are removed carefully by the use of forceps^6^.
6. Embryos are killed by decapitation.
7. Eyes are enucleated be the use of fine, curved forceps, "peeling" the eyes from the eye socket.

#### Enucleation of postnatal and adult eyes

1. Young pups are killed by decapitation, older pups and adults by cervical dislocation.
2. Up to postnatal stage P15, the time point when mice open their eyes, eye slits have to be mechanically opened by the use of forceps and enlarged by two transverse cuts of the eye lids with spring scissors.
3. Eyes are enucleated by the help of curved forceps, applying pressure to the orbit.

Note: As at postnatal day 2, the orbital bones are still cartilaginous, it is important not to apply too much pressure while trying to remove the eyes. By contrast, in the adult mice, the orbital bones are firm. Thus, in order to enucleate the eyes it is sufficient to apply pressure to the orbit without enlarging the eye slits in advance.

### Part 2: Dissection of embryonic, postnatal and adult murine retinas

#### Dissection of retinas

1. The eyes are placed in a small Petri dish with sterile PBS and the surrounding eye layers are removed under a dissection microscope.
2. To remove the outer eye layers, in postnatal stages and adult eyes the optic nerve has to be cut off by the help of spring scissors or pinched off by forceps as close to the basis as possible.
3. Turn the eye, so that the back side with the hole where the optic nerve originally resided is facing you. Enter the subretinal space between retina and pigment epithelium with the tips of two very fine forceps from the site where the optic nerve penetrated the eye layers. Note: Usually, the pigment epithelium can be easily identified by its dark colour. In some mouse mutant strains - especially in albino animals - this pigment layer may however, not be coloured and thus may not be readily to detect.
4. Remove the pigment epithelium with the attached choroid membrane and sclera by carefully tearing to either side with both forceps.
5. Peel off the layers up to the level of the cornea, then turn the eye cup to the lens side and remove the cornea together with pigment epithelium, choroid membrane and sclera, while holding up the remaining retinal cup by the other forceps.
6. Grasp the vitreous together with the small lens and while tearing with forceps, keep the retinal wholemount in place with the second forceps. Note: When dissecting embryonic eyes, make sure to completely remove the triangular, tent-like capillary plexus underneath the vitreous body together with the vitreous. In the adult eye, the vitreous needs to be grasped at the sides and care has to be taken not to pierce the vitreous with the tips of the forceps as its content is viscous and sticks to the forceps, hindering its removal.
7. For organotypic wholemount culture the retinal cups are collected in a 96-well plate containing 200 μl Dulbecco's modified eagle's medium (see below).

Note: Between dissection of individual retinas, keep the 96-well collection plate containing the culture medium in the incubator as the pH of the culture medium is triggered by CO_2_ via the carbonate system.

### Part 3: Murine organotypic retinal wholemount culture

1. Prepare 500 ml culture by weighting 7.8g Dulbecco's modified eagle's medium / nutrient mixture F-12 HAM (DMEM) and 0.6g NAHCO_3_ and dissolving both in MiIliQ water. Adjust the pH to 7.15. Add 50mg apo-transferin, 50μl putrescin (stock: 60mg/ml), 50μl sodium selenite (stock: 52μg/ml), progesterone (stock: 60μg/ml), and 2.5 ml gentamicine (200 mM) under the hood. Mix and filter through a bottle top filter. Immediately before usage add 10μl glutamine (200 mM) per ml culture medium. Note: This serum- and insulin-free medium can be stored at 4°C for up to 2 weeks and used for apoptosis induction experiments as no insulin counteracts the effects. If incubation of wholemounts for longer than 24h is desired and cell death rates will not be evaluated, insulin serum (e.g. fetal calf serum; FCS) or supplements should be added to improve survival rates.
2. Before starting the culture, the retinal wholemounts are pre-incubated for 15 min at 37°C with 200 μl warm, pH-balanced DMEM containing 0.5mg/ml hyaluronidase to pre-digest the hyaluronidase containing inner and outer limiting membrane of M ller glia cells, facilitating penetration of exogenously applied substances.
3. Retinas are transferred to a 24-well plate with as few hyaluronidase as possible and cultured as organotypic wholemounts in 2ml chemically defined Dulbecco's modified eagle medium. Note: For transferring the retinas, use a 1ml pipette and cut the pipette tip a few millimetres to widen the opening. For embryonic eyes, a 200 μl pipette tip is sufficient. The cutting edges should be smoothened by insertion and twisting of a second pipette tip. For 24-48 hrs short-term cultures, all steps can be performed at the bench, but if contamination of the cultures turns out to be a problem, one should work under the hood.
4. Cultures are maintained for 24 - 48 hrs at 37°C in a 5% CO_2_ atmosphere and e.g. subjected to pharmacological treatment with recombinant factors.

### Part 4: Dissociation of cultured retinal wholemounts

1. After the desired culture time, retinas are collected in 2ml Eppendorf tubes with round bottom containing 850 μl PBS and 50 μl bovine serum albumine (BSA; 30 mg/ml).
2. Place Eppendorf tubes with retinas into a heating block, heated up at 37°C.
3. Add 25 μl collagenase (200 U/ml) and 25 μl hyaluronidase (20mg/ml) to each Eppendorf tube and start dissociating the retinas into single cell suspension by 3 passes through a siliconized Pasteur pipette.
4. Add 10 μl trypsin (1mg/ml), wait for 3-5 min, and then slowly pipette 3-5 times up and down with siliconized Pasteur pipette to mechanically dissociate the tissue.
5. Add 10 μl DNase I (5mg/ml), again wait for 3-5 min, then slowly pipette 3-5 times up and down with siliconized Pasteur pipette. Note: The incubation time for enzymatic dissociation varies and depends on the size of the eyes and the developmental stage, respectively. Check the stage of enzymatic digestion of the tissue by gently pipetting up and down.
6. If the cell suspension is not homogenous by now but still contains major cell aggregates, add additional 10 μl trypsin and 10 μl DNase I.
7. When the cell suspension is homogeneous, digestion of the tissue is stopped by addition of 10 μl EDTA (0.5 M), Eppendorf tubes are removed from the heater and the cell suspensions are fixed for 1h by addition of 1ml fresh, ice cold 8% paraformaldehyde (PFA) at room temperature on a rotation shaker.

### Part 5: Washing of dissociated cell suspensions

1. The cell suspension is centrifuged for 5 min at 4°C and at 0.2 rcf in a cooling centrifuge.
2. The supernatant is discarded and the pellet is re-suspended in 1ml PBS containing 3mg/ml BSA.
3. After repeating these washing steps twice, the pellet is finally re-suspended in 500 μl PBS containing 3mg/ml BSA, 5mM EDTA and 0.1% sodium azide.

Note: The addition of sodium acid allow storage of the cell suspension for several days at 4°C. However, if an immunocytochemical staining will follow, do not add sodium acid to the resuspension buffer, as this results in loss of staining quality.

### Part 6: Cytospin of cell suspensions for quantitative apoptosis and proliferation analysis

1. A frosted-end microscope slide, a cytospin filter with one or two holes and a cytospin funnel are inserted into a cytospin slide clip. The slide clip is closed and positioned in the cytospin rotor.
2. The dissociated cell suspension is homogenized by gently pipetting up and down.Note: Depending on the developmental stage of the retina, the cell suspension from the dissociation procedure might need to be diluted with PBS to obtain a countable number of cells.
3. An aliquot (100 μl) of the cell suspension is applied to a cytospin funnel. Note: When pipetting the cell suspension to the funnel, the tip of the pipette should reach all the way down to the bottom of the funnel. It is important not to push through the second pressure point of the pipette as this creates air bubbles, which will be visible in the cell spot after the cytospin and hampers cell counts.
4. The cell suspension is spotted on a slide at 700 rpm for 7 min.
5. For determination of the effect of exogenously applied factors on apoptosis levels cells can be stained with 4',6-Diamidino-2-phenylindole (DAPI; 2μg/ml), mounted with fluorescent mounting medium. Changes in cell apoptosis can be determined by counting at least 1000 cells (comprising at least 10 pycnotic nuclei) in the cytospin cell spots and the cell death rate is calculated as percentage of total cells counts^3,4^. Note: Alternatively, the distribution of apoptotic nuclei can be evaluated in flatmounts^3^ or cryostat sections4 of cultured retinal wholemounts by TdT-mediated dUTP nick end labeling (TUNEL).
6. For detection of cell proliferation, BrDU (5 μM) can be added 6h before end of the culture and BrdU incorporation visualized in cytospins of cell homogenates by immunocytochemical staining using an anti-BrDU antibody (e.g. Developmental Studies Hybridoma Bank, Iowa, USA).
7. The effect of treatment on different retinal cell types can be visualized in cytospins by neuron specific antibodies like Brn3a (ganglion cell marker) or opsin (photoreceptor marker) and counterstaining with DAPI.

### Part 7: Representative Results


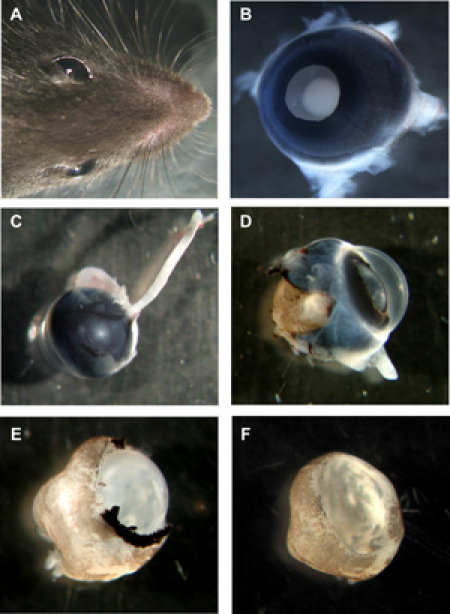
 **Figure 1: **Steps in preparation of murine organotypic retinal wholemounts**A** Head of mouse with both eyes. **B **Murine eye with lens side up, all layers still in place. **C** Murine eye from the backside with the optic nerve still attached. **D **Murine eye with sclera and pigment epithelium partially removed. **E** Murine retina with cornea, sclera and pigment epithelium completely removed, but lens and vitreous still in place. **F** Murine retinal wholemount cup with lens and vitreous removed. Please click here to see a larger version of figure 1.


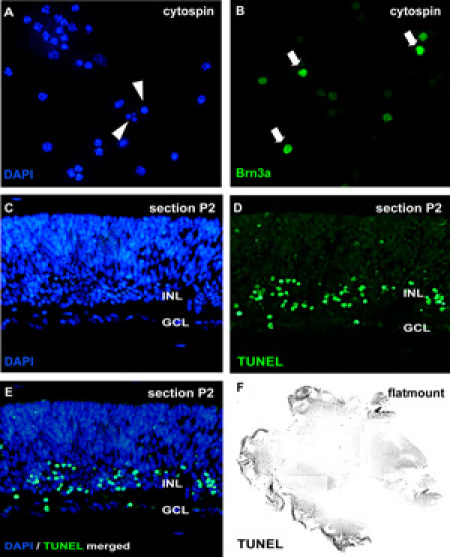
 **Figure 2: **Analysis of organotypic retinal wholemount cultures by cytospin and sectionsFor analysis of apoptosis, cytospins of dissociated cell suspensions are stained by DAPI and pycnotic nuclei can be distinguished by nuclear fragmentation or chromatin condensation **(arrowheads** in A). Alternatively, wholemount sections (C-E; murine retina postnatal day (*P*) 2) or retinal flatmount (F) can be subjected to a TUNEL assay and counterstained with DAPI (E). The effect of treatment on different retinal cell types can be visualized in cytospins by neuron specific antibodies like the ganglion cell marker Brn3a (**arrows** in B). *GCL*, ganglion cell layer; *INL,* prospective inner nuclear layer. Please click here to see a larger version of figure 2.

## Discussion

The advantage of murine organotypic retinal wholemount cultures^2-5^ over dissociation, monolayer, retinospheroid or re-aggregated 3D spheroid cultures^1^ lies in the preservation of neuronal interactions and connections, mimicking the *in vivo* situation. In comparison to former reports^2^, our video article provides a detailed demonstration of the peculiarities in enucleation of murine eyes and dissection of retinas of different developmental stages including removal of lens and vitreous body without damaging the retina. The removal of the lens and vitreous is essential for pharmacological manipulations as both hinder the access of substances to the retinal layers. In contrast to other reported murine explant culture systems, we do not use supportive materials, e.g. polycarbonate membranes^2^, for our organotypic culture, but culture the retinal wholemount cups free floating, additionally facilitating the accessibility for exogenously applied substances^3-5^.

Using a chemically defined, serum- and supplement-free culture medium without insulin allows only for short-time culture (24 - 48 hrs) but counterbalancing effects of insulin on apoptosis levels^3^ or growth factor-mimicking effects of FCS and supplements are avoided.

Most apoptosis and proliferations studies use MTT assays or FACS analysis for quantification of effects. We, however, provide a step by step demonstration of dissociation of cultured retinas for quantitative apoptosis and proliferation****analysis by cytospin. As far as our experience goes, manual counting of nuclei in DAPI or BrDU stained cell spots - although tedious and time consuming - is the most accurate method for quantifying retinal apoptosis and proliferation, especially in murine retinas.

## References

[B0] Rieke M, Gottwald E, Weibezahn K-F, Layer PG (2008). Tissue reconstruction in 3D-spheroids from rodent retina in a motion-free, bioreactor-based microstructure. Lab. Chip.

[B1] Donovan SL, Dyer MA (2006). Preparation and square wave electroporation of retinal explant cultures. Nature Protocols.

[B2] Duenker N, Valenciano AI, Franke A, Hernandez-Sanchez C, Dressel R, Behrendt M, de Pablo F, Krieglstein K, de la Rosa EJ (2005). Balance of pro-apoptotic transforming growth factor-beta and anti-apoptotic insulin effects in the control of cell death in the postnatal mouse retina. Eur. J. Neurosci.

[B3] Franke AG, Gubbe C, Beier M, Duenker N (2005). Transforming growth factors beta and Bone morphogenetic proteins: Cooperative players in chick and murine programmed retinal cell death. J. Comp. Neurol.

[B4] de la Rosa EJ, Díaz B, De Pablo F (1998). Organoculture of the chick embryonic neuroretina. Curr. Top. Dev. Biol.

[B5] Dohle DS, Pasa SD, Gustmann S, Laub M, Wissler JH, Jennissen HP, Duenker N (2009). Chick ex ovo culture and ex ovo CAM assay: How it really works. J Vis Exp.

